# Inhibition of avian tumor virus replication by CCCH-type zinc finger antiviral protein

**DOI:** 10.18632/oncotarget.19378

**Published:** 2017-07-19

**Authors:** Mingjun Zhu, Xiaoqian Ma, Xiyao Cui, Jing Zhou, Chengui Li, Libo Huang, Yingli Shang, Ziqiang Cheng

**Affiliations:** ^1^ College of Veterinary Medicine, Shandong Agricultural University, Tai’an, 271018, China

**Keywords:** chicken CCCH type zinc finger antiviral protein, avian leukosis virus subgroup J, overexpression, RNAi

## Abstract

CCCH type zinc finger antiviral protein (ZAP) is a host restriction factor that inhibits the replication of a variety of viruses in mammals. However, little is known about its antiviral activity on avian tumor virus. Avian leukosis virus subgroup J (ALV-J), an oncogenic retrovirus, induces myelocytomas and various other tumors in meat and egg type chickens. Here, we identified a chicken ZAP (chZAP) that increased at early stage, and subsequently decreased after infection of ALV-J in DF-1 cells, indicating the inducible feature of the endogenous chZAP. To demonstrate the inhibitory effect on ALV-J replication by chZAP, we expressed exogenous chZAP by lentivirus based vectors in DF-1 cells that infected by ALV-J. The result showed that overexpression of chZAP significantly inhibited ALV-J replication at both mRNA level and protein level. Consequently, knockdown of endogenous chZAP by RNAi facilitated ALV-J replication in DF-1 cells. Further, we demonstrated that chZAP interacts with SU protein (encode by gp85 gene) of ALV-J in cytoplasm. Taken together, our results demonstrated that chZAP inhibits ALV-J by both mRNA and protein pathway and it may shed light on a novel antiviral approach in poultry.

## INTRODUCTION

Host restriction factors play critical roles in control of retrovirus replication [1]. In recent years, a number of powerful host restriction factors that specifically inhibit retrovirus replication have been identified in mammals including APOBEC3, TRIM5, KAP1/TRIM28/ZFP809, SAMHD1, Tetherin and the CCCH type zinc finger antiviral protein (ZAP) [2–7]. Most of the restriction factors were identified from non-permissive cells, in which viral replication is severely restricted, indicating that restriction factors constitute novel aspects of intrinsic immunity. In addition, restriction factors can display hallmarks of positive genetic selection during evolution which benefits the host in settings of host-pathogen conflicts [8]. The findings of a serial of host restriction factors have not only provided us comprehensive understanding of the restriction factors, but also offered us new strategies to study the mechanisms of retrovirus-host interaction.

CCCH type ZAP contains four zinc finger motifs at the N terminus and each has a cysteine–histidine repeat in a cys–cys–cys–his (CCCH) configuration. ZAP was initially identified in rat as a host restriction factor that prevents cells from infection with the moloney murine leukemia virus (MMLV) [3]. In addition to MMLV, ZAP has an extensive antiviral spectrum that includes alphaviruses, such as Sindbis virus, Ross River virus, xenotropic murine leukemia virus-related virus and human immunodeficiency virus; filoviruses, such as Ebola virus and Marburg virus [9–11] and hepadnaviridae, such as hepatitis B virus [12]. Although ZAP is a host restriction factor of the innate immune system, ZAP does not induce a universal antiviral state because some viruses, such as herpes simplex virus type 1 and yellow fever virus, can replicate normally in ZAP-overexpressing cells [9]. ZAP reduces the level of mRNA in the cytoplasm to suppress target virus infection at the posttranscriptional stage, whereas ZAP does not inhibit the early stage of virus infection [3]. ZAP recognizes the target virus transcripts via its CCCH type zinc-finger domains and binds to RNA helicases and components of the exosome to induce the degradation of virus transcripts [13–17].

Avian leukosis virus subgroup J (ALV-J), an oncogenic retrovirus belongs to the alpharetroviral genus of the family *Retroviridae*, is a causative agent of immunosuppression and myelocytomas and various other tumors in chicken. ALV-J was isolated by Payne in the UK in 1991 from meat-type broilers [18]. After that, this disease caused by ALV-J has spread rapidly worldwide and has become a major problem in the meat-type and layer-type chicken industry. Feng observed that ZC3HAV1 (chZAP) mRNA showed a two-fold up-regulation in spleen at day 4 post infection of ALV-J, indicating the association between the innate immune factor of chZAP and ALV-J [19]. However, little is known about the antiviral activity of chZAP on ALV-J.

## RESULTS

### Endogenous chZAP expression was induced by infection of ALV-J

**Figure 1:**
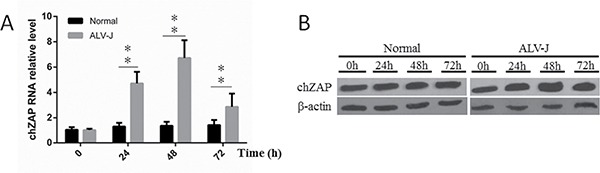
Endogenous chZAP expression was induced by infection of ALV-J At 24 h and 48 h post infection with ALV-J, the chZAP mRNA level (**A**) and protein expression level (**B**) of infected cells was significantly (*P < 0.05) higher than the uninfected normal control cells. At 72 h post infection, the chZAP level decreased significantly (**P < 0.01). Results shown are mean ± SEM of three independent experiments.

To understand whether endogenous chZAP expression was affected by ALV-J infection in cells, we examined the endogenous chZAP changes by qPCR, western blot before and after infection of ALV-J in DF-1 cells. As shown in Figure 1A, cellular endogenous RNA level of chZAP was extremely significant increased (*P* < 0.01) at 24 h and 48 h, and then was decreased (*P* < 0.01) at 72 h post infection of ALV-J. Accordingly, protein level of endogenous chZAP showed same trend (Figure 1B).

### Overexpression of chZAP suppressed ALV-J replication

**Figure 2:**
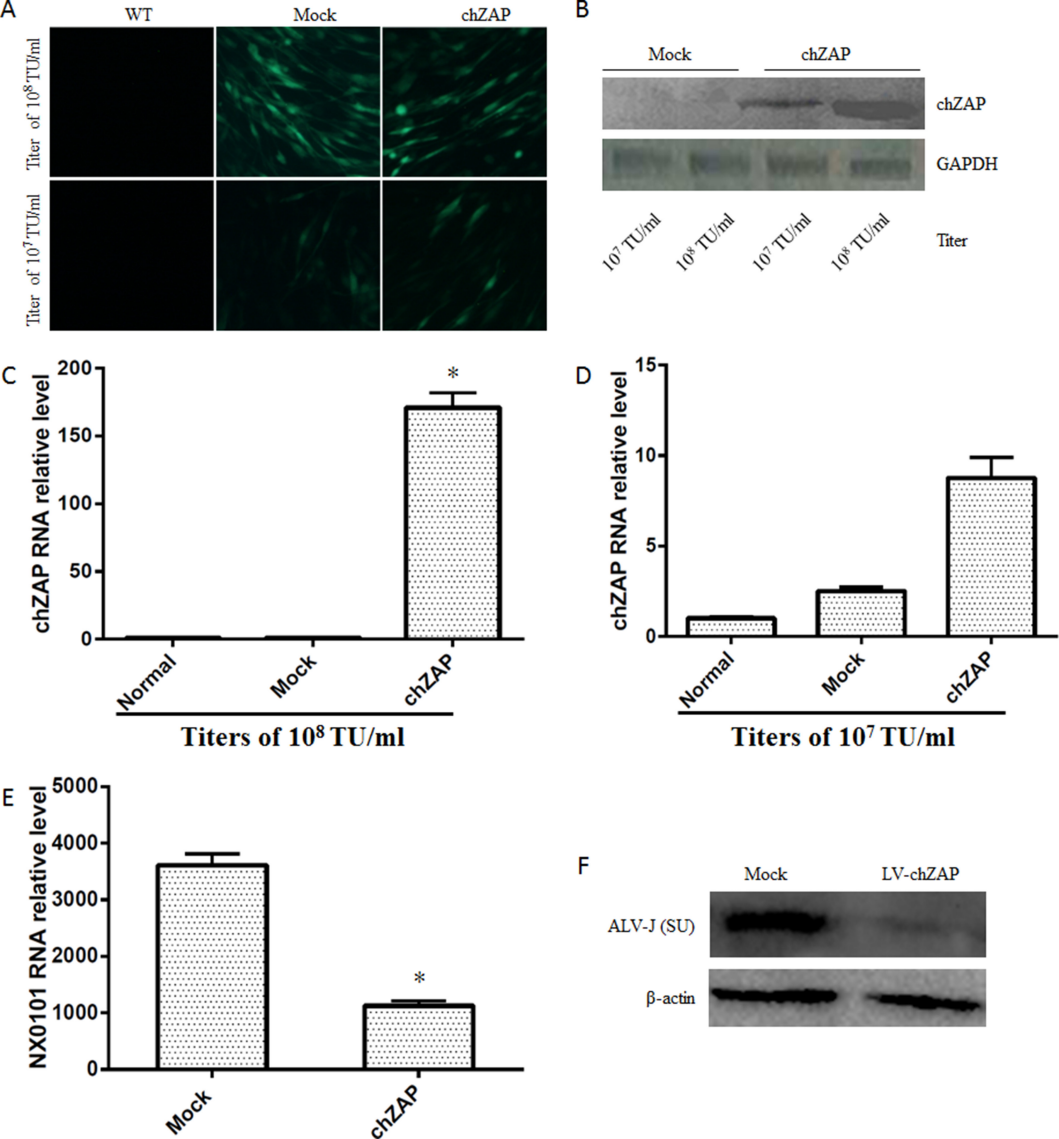
Overexpression of chZAP suppressed ALV-J replication At 72 h post transduction of lentivirus-chZAP, DF-1 cells were infected with 100 μl 10^3^ TCID_50_ ALV-J. The cells were harvested at 72 h post infection with ALV-J, and the viral RNA was analyzed by qPCR. Overexpression of chZAP (**A, B, C** and **D**) significantly inhibits ALV-J replication (*p < 0.05) at the titer of 1 × 10^8^ TU/ml (**E** and **F**) while not 1 × 10^7^ TU/ml. Data is representative of three independent experiments and statistical significance was calculated by one-way analysis of variance (ANOVA) with Tukey's test for multiple comparisons comparing all samples to each other.

Two titers of lentivirus-chZAP (10^7^ TU/ml and 10^8^ TU/ml) were used to transfect the DF-1 cells. At 72 post transfection, the green fluorescence of GFP observed under fluorescent microscope (Figure 2A) and the expression levels of chZAP measured by western blot (Figure 2B) and qPCR (Figure 2C and 2D) indicate that chZAP significantly expressed in DF-1 cells under transfection titers of 10^8^ TU/ml. Overexpression of chZAP transfected with titer of 10^8^ TU/ml led to a significant reduction of mRNA (*p* < 0.05) (Figure 2E) and protein expression (Figure 2F) of ALV-J in DF-1 cells.

### Down-regulation of endogenous chZAP facilitated ALV-J replication

**Figure 3:**
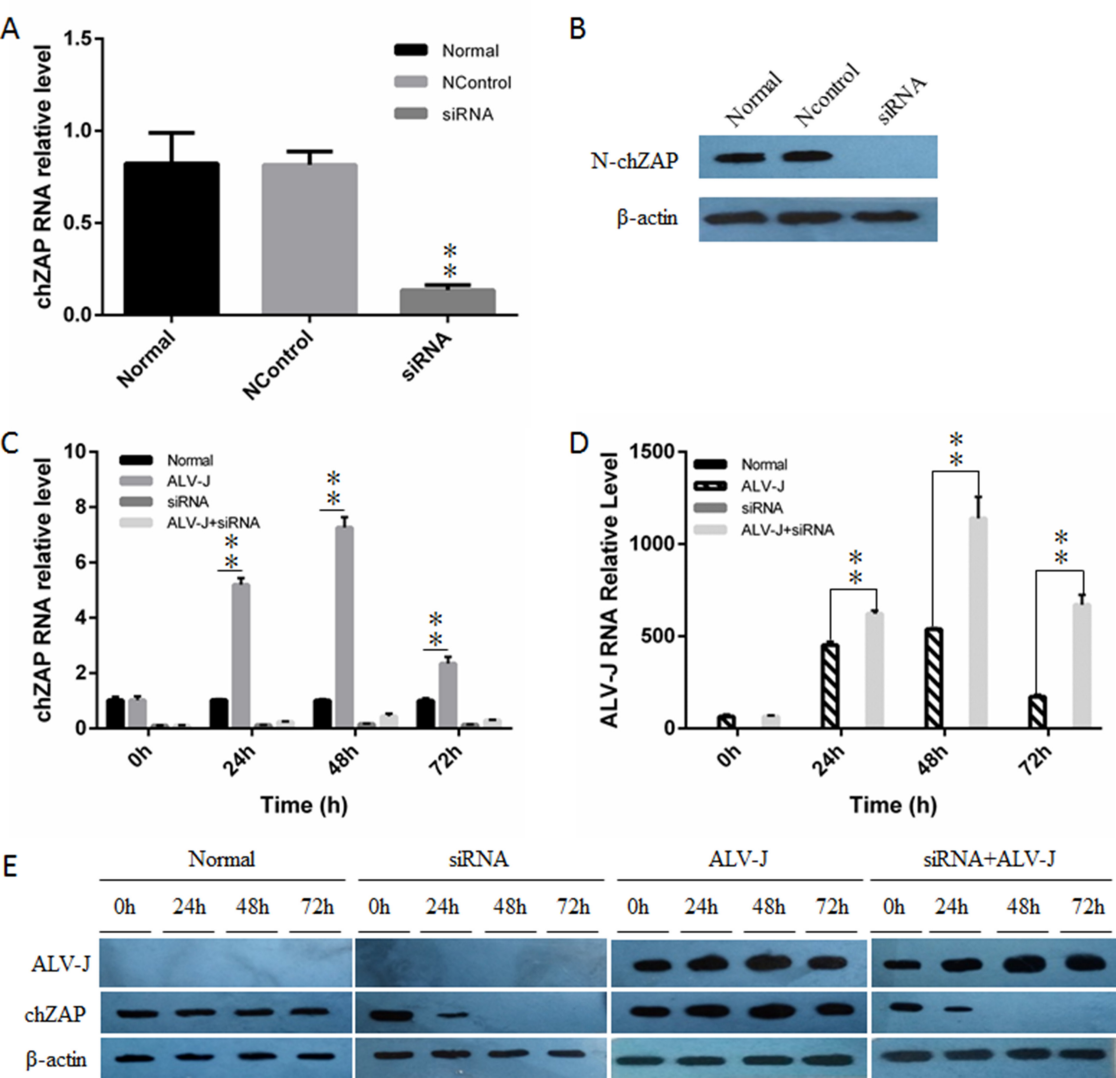
Knockdown of endogenous chZAP facilitated ALV-J replication chZAP-specific siRNA significantly knocked down RNA level (**A** and **C**) and protein expression level (**B** and **E**) of chZAP. Knockdown of endogenous chZAP expression significantly increased the mRNA level (**P < 0.01) (**D**) and env expression level (E) of ALV-J in DF-1 cells. Data is representative of three independent experiments and statistical significance was calculated by one-way analysis of variance (ANOVA) with Tukey's test for multiple comparisons comparing all samples to each other.

To confirm the inhibitory role of chZAP on ALV-J, we knocked down the basal expression of chZAP in DF-1 cells by using chZAP-specific siRNAs, and then infected with ALV-J (10^3^ TCID_50_). The result showed that chZAP-specific siRNAs efficiently reduced the basal levels of chZAP expression to more than 70% of its original expression (Figure 3A and 3B). The levels of chZAP expression kept low stably (Figure 3C and 3E) after RNAi and led to a significant increase of ALV-J mRNA (Figure 3D) (*P* < 0.01) and env protein expression (Figure 3E). The result suggested that the basal level of chZAP is a robust host restriction factor for ALV-J.

### chZAP interacted with SU protein of ALV-J in cytoplasm

**Figure 4:**
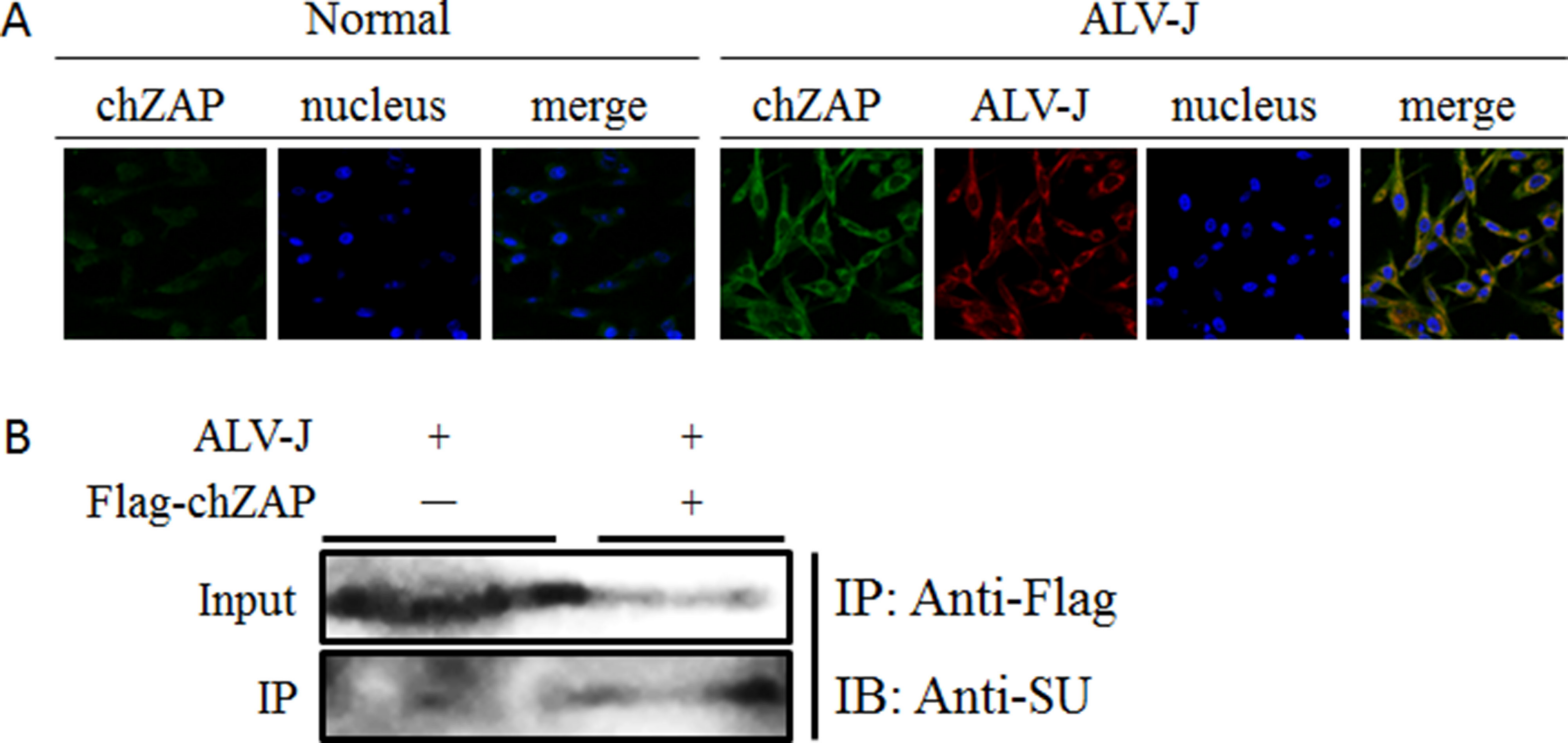
chZAP interacted with SU of ALV-J in cytoplasm (**A**) ALV-J and endogenous chZAP are both present in cytoplasm of DF-1 cells. At 48 h post infection with ALV-J, the protein expression level of chZAP of infected cells was very significantly higher than the uninfected normal control cells. The merged yellow fluorescence indicated the interaction between chZAP and ALV-J. (**B**) chZAP interacted with SU. DF-1 cells were transfected with empty vector (chZAP-) or LV-chZAP expressing flag-chZAP (chZAP+). The cells were harvested at 72 h post infection with ALV-J. Lysates were immunoprecipitated with anti-Flag antibody and detected by western blotting using anti-SU antibody.

To probe how chZAP interferes with the protein level of ALV-J, we infected DF-1 cells with ALV-J after transfected with Lentivirus-chZAP (10^8^ TU/ml) and investigated the interactions between chZAP and ALV-J by confocal laser scanning microscopy (CLSM) and co-immunoprecipitation (Co-IP). CLSM result further confirmed that ALV-J can induce chZAP expression, suggesting the inducible activation of chZAP and its inhibitory role for ALV-J. Moreover, the merged yellow fluorescence of ALV-J and chZAP indicated the interaction between SU of ALV-J and chZAP and the interaction occurred in the cytoplasm (Figure 4A). Correspondingly, Co-IP result confirmed the interaction between SU and chZAP (Figure 4B).

## DISCUSSION

Viral pathogenesis is determined by the interaction between viruses and hosts. They both change dynamically in multiple ways in response to each other. In host, the innate immunity plays an important role at the initial infection of virus [20]. The innate immunity sets up a first-line defense to prevent the host from viral infection. In principle, the innate immunity consists of many viral inhibitory factors that intervene against the virus at every step of replication. Such inhibitory factors are all belong to host restriction factors. Among them, CCCH-type ZAP showed a specific antiviral activity in mammals. Chicken CCCH-type ZAP (chZAP) gene was first predicted in a high-quality library of chicken bursal lymphocytes cDNA [21]. However, little is known about its role of antiviral activity on avian retrovirus.

To understand the interaction between chZAP and ALV-J, firstly, we detected the basal chZAP expression in normal and ALV-J infected DF-1 cells. Interestingly, ALV-J infection induced increasing expression of endogenous chZAP at the early time point (24 h and 48 h), indicating that chZAP is an inducible factor. However, chZAP was down-regulation at 72 h post infection of ALV-J, suggesting that there exist the interaction between the chZAP and ALV-J. Next, we want to know if the decreasing of chZAP was directly caused by ALV-J. To answer this question, we packaged the lentivirus-chZAP to overexpress exogenous chZAP in cultured DF-1 cells. Liu had demonstrated that rat ZAP is predominantly localized in the cytoplasm at steady state but shuttles between the nucleus [22]. Shuttling proteins are key factors in conveying information on nuclear and cytoplasmic activities within the cell [23]. Subsequently, we transfected the lentivirus-chZAP into DF-1 cells that infected by ALV-J. qPCR result showed that the overexpressed chZAP significantly inhibited ALV-J replication, and the antiviral activity of chZAP against ALV-J was dependent on the expression level of chZAP. Knockdown of endogenous chZAP together with increasing ALV-J replication demonstrated the inhibition of ALV-J by chZAP, indicating the robust host restriction factor of chZAP for ALV-J. Previous studies suggested that ZAP interacts with target RNA of virus through its zinc finger motifs [12]. However, there have been no reports of interactions between proteins for virus and ZAP. Results from our study suggested that chZAP interacts with SU protein of ALV-J. We demonstrated that chZAP exhibits its antiviral activity in cytoplasm by interaction with mRNA and SU protein of ALV-J.

Taken together, the work reported here has identified an antiviral protein of chZAP as a host restriction factor that inhibits ALV-J replication by mRNA and protein pathway. chZAP-mediated antiviral function may be used to develop novel antiviral strategies in poultry.

## MATERIALS AND METHODS

### Cells and virus

DF-1 cells and 293T cells were maintained in Dulbecco's Modified Eagle's Medium (DMEM) supplemented with 10% fetal bovine serum (FBS) (GIBCO, America), respectively. The stock NX0101 strain of ALV-J that isolated from a broiler breeder was maintained in our laboratory. The TCID_50_ of NX0101 strain was determined by limiting dilution in DF-1 culture.

### Quantitative real time RT-PCR (qPCR)

DF-1 cells in DMEM with 10% FBS were seeded in a 6-well plate at 1 × 10^6^ cells per well. When the confluence of DF-1 cells reached 80%, a volume of 100 μl 10^3^ TCID_50_ of ALV-J was added to the culture medium. Total RNA was extracted and reverse transcripted to cDNA. The 20 μl reaction system contained 1 μl cDNA, 0.4 μl Rox Reference Dye II (50X), 10 μl SYBR^®^ Premix Ex Taq^TM^ (TaKaRa), and 8 pmol primers for ALV-J (forward: 5′-TGCGTGCGTGGTTATTATTTC-3′ and reverse: 5′-AATGGTGAGGTCGCTGACTGT-3′). The GAPDH mRNA level was the internal control. The reactions were run on an Applied Biosystems 7500 Prism real-time PCR machine using the SYBR^®^ Premix Ex Taq^TM^ (TaKaRa) for ALV-J with the following steps: (i) 30 s at 95°C and (ii) 40 cycles of 1 cycle consisting of 5 s at 95°C and 34 s at 60°C, followed by melting curve analysis. The chZAP was amplified with the following primers: forward: 5′ACCAGTGCTGAGAACAAA3′ and reverse: 5′CATCAGGAAGGAGGAAAG3′. The 2^-ΔΔCt^ method was used to analyze the results [24].

### Lentivirus-chZAP package

GV virus carrier which was recombined with chZAP gene and enhanced green fluorescent protein (EGFP) gene, pHelper 1.0 carrier and pHelper 2.0 carrier were extracted from DH5α and purified without endotoxin. GV virus carrier contains essential elements 5′LTR and 3′LTR as well as other auxiliary elements of HIV. pHelper 1.0 carrier contains gag gene encoding the major structural protein of the HIV, pol gene encoding virus-specific enzyme, and rev gene encoding regulators for gag and pol gene expression. pHelper 2.0 carrier contains VSV-G gene which provides membrane proteins for viral packaging. The three carriers were co-transducted to 293T cells followed by the manufacturer's instructions to obtain the lentivirus-chZAP recombinant vector. The titer of the lentivirus-chZAP recombinant vector was determined by limiting dilution in 293T cell culture (Shanghai GeneChem Co., Ltd). The optimal transfection titer in DF-1 cells for the recombinant vector is 10^7^~10^8^ TU/ml. Polybrene (5 μg/ml) was added to enhance the transfection. The lentivirus-chZAP recombinant vector which contains EGFP and chZAP genes were successfully transfected into DF-1 cells, then integrated into the genome to be high level expression. The expression time procession of the lentivirus-chZAP recombinant vector was determined by the expression of EGFP observing under a fluorescence microscope, and the mRNA level of chZAP was tested by qPCR.

### Measurement of ALV-J replication in chZAP-overexpressed DF-1 cells

To evaluate the antiviral activity of chZAP, we used overexpressed recombinant lentivirus-chZAP to inhibit ALV-J replication in DF-1 cells. DF-1 cells were seeded at 2 × 10^5^ cells per well in a 24-well plate and transfected the following day with lentivirus-chZAP recombinant vector at titers of 10^7^ TU/ml or 10^8^ TU/ml. At 72 h post-transfection, green fluorescence of the recombinant vector was observed. A volume of 100 μl 10^3^ TCID_50_ of ALV-J was added to the culture medium and cultured for 72 h. The cells were harvested at the terminal time points of this experiment and tested by qPCR.

### RNA interference (RNAi)

chZAP siRNA (Cat. siG151014100702) and control siRNA (Cat. siN05815122147) were synthesized by Ruibo Biotechnology, Inc (Jinan, China). To confirm the inhibition of ALV-J by chZAP, chZAP-specific siRNA was transducted into DF-1 cells, and then infected ALV-J. siRNA transduction (50 nM) was performed by Lipofectamine 2000 according to manufacturer's directions. The RNA and protein level of chZAP and env protein of ALV-J were tested by qPCR and western blot, respectively at 0 h, 24 h, 48 h and 72 h post infection of ALV-J.

### Western blot

Transduced DF-1 cells were washed once with PBS buffer, lysed in cell lysis buffer (Beyotime) and incubated on ice for 5 minutes. The lysates were resuspended in SDS loading buffer, boiled for 5 min, loaded and run on a 12% SDS-PAGE, and then transferred onto a nitrocellulose membrane (Solarbio). The nitrocellulose membrane was blocked with 5% skimmed milk at 4°C overnight and probed with a polyclonal antibody against chZAP at a 1:200 dilution, followed by horseradish peroxidase (HRP)-conjugated goat anti-rabbit secondary antibody (Bioss) at a 1:3000 dilution. The beta-actin was as reference control. Detection was performed with Enhanced HRP-DAB Chromogenic Substrate Kit (Tiangen) according to the manufacturer's instructions.

### Confocal laser scanning microscopy (CLSM)

DF-1 cells were seeded in confocal dishes the day prior to infection and maintained in DMEM with 10% FBS. When the cell confluence reached 80%, the cells were infected with 200 μl NX0101 strain of ALV-J at 10^3^ TCID_50_ per dish. The cells were washed three times with PBS (137 mM NaCl, 2.7 mM KCl, 10 mM Na_2_HPO_4_, and 2 mM KH_2_PO_4_) and were fixed in fixative (1 ml of fixative contains 600 μl of acetone plus 400 μl of alcohol) at 0 h, 24 h, 48 h and 72 h post infection. 7 minutes later, the fixative was removed and the cells were washed three times with PBS. Then, the cells were incubated with primary anti-chZAP (1:50) or anti-SU (1:200) antibody at 4°C for 10 h. After that, the cells were washed three times with PBS, and incubated with HRP-labeled Goat anti-rabbit IgG (H+L) (1:5000) and HRP-labeled Goat anti-mouse IgG (H+L) (1:5000) at 37°C for 1.5 h. Then, the cells were washed three with PBS. Nucleuses were coloring with DAPI at 37°C for 5 minute, and then the cells were washed three with PBS again. Finally, the cells with 1 ml of 50% glycerol were observed in CLSM immediately.

### Co-immunoprecipitation assay

DF-1 cells were seeded in 12 holes plate. Cells were infected with ALV-J (10^3^ TCID^50^) after transfected by lentivirus-chZAP (10^8^ TU/ml). The cells were harvested at 72 h post infection and lysed in lysis buffer (20 Mm Tris, PH 7.5, 150 mM NaCl, 1% Triton X-100, 1 mM EDTA, 10 μg/ml aprotinin, 10 μg/ml leupeptin, 1 mM phenylmethylsulfonyl fluoride). Cell lysate was incubated with 1 μg of the indicated antibody and 25 μl carboxyl beads (2 mg/ml) for 8 h at 4°C. Immunoprecipitates were washed three times with TBST, re-suspended in SDS loading buffer, boiled for 5 min and analyzed by western blot with indicated antibody.

### Statistical analysis

The following programs were used for statistical analysis: SPSS Statistics, Graphpad prism6.

### Accession number

The sequence of chZAP gene was deposited in GenBank, and the accession number is KJ675563.
